# Supplementary material for: Trends in dental expenditures in Japan with a universal health insurance system
Source: PLoS One. 2023 Oct 5;18(10):e0292547. doi: 10.1371/journal.pone.0292547 (PMC10553203; doi:10.1371/journal.pone.0292547)
Supplement: S1 Table — (DOCX) [file pone.0292547.s001.docx]

**S1 Table. Amount and proportion of services per year**

| **Year** | **Initial- and repeat-consultation fee (A)** | | **Medical management (B)** | | **At-home treatment (C)** | | **Tests (D)** | | **Diagnostic imaging (E)** | | **Drug administration (F)** | | **Injection (G)** | | **Rehabilitation (H)** | | **Treatment (I)** | | **Surgery (J)** | | **Anaesthesia (K)** | | **Radiotherapy (L)** | | **Crown restoration and prosthesis (M)** | | **Orthodontic treatment (N)** | | **Pathological diagnosis (O)** | | **Hospitalisation fee** | | **Others** | |
| --- | --- | --- | --- | --- | --- | --- | --- | --- | --- | --- | --- | --- | --- | --- | --- | --- | --- | --- | --- | --- | --- | --- | --- | --- | --- | --- | --- | --- | --- | --- | --- | --- | --- | --- |
|  | **Amount** | **%** | **Amount** | **%** | **Amount** | **%** | **Amount** | **%** | **Amount** | **%** | **Amount** | **%** | **Amount** | **%** | **Amount** | **%** | **Amount** | **%** | **Amount** | **%** | **Amount** | **%** | **Amount** | **%** | **Amount** | **%** | **Amount** | **%** | **Amount** | **%** | **Amount** | **%** | **Amount** | **%** |
|  | **(1 trillion yen [≈ 10 billion US dollars])** |  | **(1 trillion yen [≈ 10 billion US dollars])** |  | **(1 trillion yen [≈ 10 billion US dollars])** |  | **(1 trillion yen [≈ 10 billion US dollars])** |  | **(1 trillion yen [≈ 10 billion US dollars])** |  | **(1 trillion yen [≈ 10 billion US dollars])** |  | **(1 trillion yen [≈ 10 billion US dollars])** |  | **(1 trillion yen [≈ 10 billion US dollars])** |  | **(1 trillion yen [≈ 10 billion US dollars])** |  | **(1 trillion yen [≈ 10 billion US dollars])** |  | **(1 trillion yen [≈ 10 billion US dollars])** |  | **(1 trillion yen [≈ 10 billion US dollars])** |  | **(1 trillion yen [≈ 10 billion US dollars])** |  | **(1 trillion yen [≈ 10 billion US dollars])** |  | **(1 trillion yen [≈ 10 billion US dollars])** |  | **(1 trillion yen [≈ 10 billion US dollars])** |  | **(1 trillion yen [≈ 10 billion US dollars])** |  |
| 1996 | 0.28579 | 10.8 | 0.12626 | 4.8 | 0.00883 | 0.3 | 0.12695 | 4.8 | 0.09406 | 3.6 | 0.04949 | 1.9 | 0.00378 | 0.1 | 0.00017 | 0.0 | 0.49579 | 18.7 | 0.10095 | 3.8 | 0.00741 | 0.3 | 0.00072 | 0.0 | 1.33351 | 50.3 | 0.00085 | 0.0 | - | - | 0.01441 | 0.5 | 0.00001 | 0.0 |
| 1997 | 0.27322 | 10.5 | 0.14445 | 5.6 | 0.01053 | 0.4 | 0.11940 | 4.6 | 0.09540 | 3.7 | 0.04909 | 1.9 | 0.00333 | 0.1 | 0.00056 | 0.0 | 0.47701 | 18.4 | 0.09724 | 3.7 | 0.00682 | 0.3 | 0.00044 | 0.0 | 1.30012 | 50.1 | 0.00403 | 0.2 | - | - | 0.01242 | 0.5 | 0.00000 | 0.0 |
| 1998 | 0.28723 | 11.2 | 0.15881 | 6.2 | 0.01289 | 0.5 | 0.11398 | 4.4 | 0.08579 | 3.3 | 0.04606 | 1.8 | 0.00308 | 0.1 | 0.00045 | 0.0 | 0.48119 | 18.8 | 0.09966 | 3.9 | 0.00640 | 0.2 | 0.00049 | 0.0 | 1.25006 | 48.8 | 0.00164 | 0.1 | - | - | 0.01554 | 0.6 | 0.00001 | 0.0 |
| 1999 | 0.29425 | 11.3 | 0.17615 | 6.8 | 0.01266 | 0.5 | 0.12657 | 4.9 | 0.09513 | 3.7 | 0.04833 | 1.9 | 0.00257 | 0.1 | 0.00028 | 0.0 | 0.49774 | 19.2 | 0.09767 | 3.8 | 0.00686 | 0.3 | 0.00024 | 0.0 | 1.22300 | 47.1 | 0.00052 | 0.0 | - | - | 0.01365 | 0.5 | 0.00000 | 0.0 |
| 2000 | 0.30598 | 11.6 | 0.18492 | 7.0 | 0.00908 | 0.3 | 0.13294 | 5.1 | 0.08829 | 3.4 | 0.04886 | 1.9 | 0.00287 | 0.1 | 0.00079 | 0.0 | 0.40060 | 15.2 | 0.10455 | 4.0 | 0.00865 | 0.3 | 0.00025 | 0.0 | 1.31702 | 50.1 | 0.00352 | 0.1 | - | - | 0.01952 | 0.7 | 0.00000 | 0.0 |
| 2001 | 0.30945 | 11.5 | 0.18996 | 7.1 | 0.01127 | 0.4 | 0.12710 | 4.7 | 0.08474 | 3.1 | 0.04991 | 1.9 | 0.00234 | 0.1 | 0.00030 | 0.0 | 0.40324 | 15.0 | 0.10108 | 3.8 | 0.00675 | 0.3 | 0.00022 | 0.0 | 1.39139 | 51.7 | 0.00207 | 0.1 | - | - | 0.01313 | 0.5 | 0.00000 | 0.0 |
| 2002 | 0.35032 | 13.0 | 0.19809 | 7.3 | 0.01443 | 0.5 | 0.14659 | 5.4 | 0.09601 | 3.6 | 0.04614 | 1.7 | 0.00227 | 0.1 | 0.00069 | 0.0 | 0.41595 | 15.4 | 0.10277 | 3.8 | 0.00654 | 0.2 | 0.00028 | 0.0 | 1.30228 | 48.2 | 0.00092 | 0.0 | - | - | 0.01767 | 0.7 | 0.00000 | 0.0 |
| 2003 | 0.34417 | 13.0 | 0.19219 | 7.2 | 0.03115 | 1.2 | 0.13991 | 5.3 | 0.08757 | 3.3 | 0.04713 | 1.8 | 0.00283 | 0.1 | 0.00044 | 0.0 | 0.41442 | 15.6 | 0.10043 | 3.8 | 0.00735 | 0.3 | 0.00024 | 0.0 | 1.26926 | 47.8 | 0.00079 | 0.0 | - | - | 0.01918 | 0.7 | 0.00000 | 0.0 |
| 2004 | 0.36399 | 13.7 | 0.21764 | 8.2 | 0.02544 | 1.0 | 0.14375 | 5.4 | 0.09422 | 3.5 | 0.05045 | 1.9 | 0.00240 | 0.1 | 0.00032 | 0.0 | 0.40908 | 15.4 | 0.10104 | 3.8 | 0.00643 | 0.2 | 0.00042 | 0.0 | 1.22560 | 46.1 | 0.00338 | 0.1 | - | - | 0.01312 | 0.5 | 0.00000 | 0.0 |
| 2005 | 0.38379 | 14.2 | 0.22492 | 8.3 | 0.01468 | 0.5 | 0.15025 | 5.6 | 0.09713 | 3.6 | 0.05040 | 1.9 | 0.00307 | 0.1 | 0.00034 | 0.0 | 0.43827 | 16.2 | 0.09011 | 3.3 | 0.00714 | 0.3 | 0.00036 | 0.0 | 1.22884 | 45.4 | 0.00185 | 0.1 | - | - | 0.01536 | 0.6 | 0.00000 | 0.0 |
| 2006 | 0.30585 | 11.7 | 0.26495 | 10.1 | 0.01896 | 0.7 | 0.16330 | 6.2 | 0.09758 | 3.7 | 0.04663 | 1.8 | 0.00240 | 0.1 | 0.00019 | 0.0 | 0.41433 | 15.8 | 0.08774 | 3.3 | 0.00615 | 0.2 | 0.00032 | 0.0 | 1.19529 | 45.6 | 0.00286 | 0.1 | - | - | 0.01533 | 0.6 | 0.00001 | 0.0 |
| 2007 | 0.29838 | 11.4 | 0.24621 | 9.4 | 0.01946 | 0.7 | 0.15566 | 5.9 | 0.09588 | 3.7 | 0.04584 | 1.8 | 0.00224 | 0.1 | 0.00024 | 0.0 | 0.44084 | 16.8 | 0.08068 | 3.1 | 0.00563 | 0.2 | 0.00031 | 0.0 | 1.20971 | 46.2 | 0.00078 | 0.0 | - | - | 0.01553 | 0.6 | 0.00001 | 0.0 |
| 2008 | 0.30915 | 11.6 | 0.32850 | 12.3 | 0.02318 | 0.9 | 0.16688 | 6.3 | 0.09950 | 3.7 | 0.04506 | 1.7 | 0.00248 | 0.1 | 0.00043 | 0.0 | 0.43508 | 16.3 | 0.07848 | 2.9 | 0.00707 | 0.3 | 0.00061 | 0.0 | 1.14070 | 42.8 | 0.00476 | 0.2 | 0.00138 | 0.1 | 0.01966 | 0.7 | - | - |
| 2009 | 0.29839 | 11.1 | 0.33557 | 12.5 | 0.05647 | 2.1 | 0.16801 | 6.3 | 0.09754 | 3.6 | 0.04589 | 1.7 | 0.00221 | 0.1 | 0.00055 | 0.0 | 0.42469 | 15.9 | 0.08383 | 3.1 | 0.00709 | 0.3 | 0.00040 | 0.0 | 1.12915 | 42.1 | 0.00466 | 0.2 | 0.00157 | 0.1 | 0.02324 | 0.9 | - | - |
| 2010 | 0.34039 | 12.4 | 0.33909 | 12.4 | 0.06524 | 2.4 | 0.16795 | 6.1 | 0.10024 | 3.7 | 0.04581 | 1.7 | 0.00216 | 0.1 | 0.00115 | 0.0 | 0.44753 | 16.3 | 0.08466 | 3.1 | 0.00693 | 0.3 | 0.00057 | 0.0 | 1.11490 | 40.6 | 0.00346 | 0.1 | 0.00204 | 0.1 | 0.02262 | 0.8 | - | - |
| 2011 | 0.35614 | 12.6 | 0.36705 | 13.0 | 0.05864 | 2.1 | 0.18355 | 6.5 | 0.10485 | 3.7 | 0.04333 | 1.5 | 0.00166 | 0.1 | 0.00078 | 0.0 | 0.46867 | 16.6 | 0.08530 | 3.0 | 0.00757 | 0.3 | 0.00051 | 0.0 | 1.12812 | 39.8 | 0.00523 | 0.2 | 0.00134 | 0.0 | 0.01869 | 0.7 | - | - |
| 2012 | 0.35586 | 12.4 | 0.31246 | 10.9 | 0.07535 | 2.6 | 0.17587 | 6.1 | 0.10959 | 3.8 | 0.04193 | 1.5 | 0.00051 | 0.0 | 0.00152 | 0.1 | 0.51987 | 18.1 | 0.08200 | 2.9 | 0.00719 | 0.3 | 0.00045 | 0.0 | 1.16582 | 40.6 | 0.00207 | 0.1 | 0.00153 | 0.1 | 0.01912 | 0.7 | - | - |
| 2013 | 0.35876 | 12.4 | 0.32472 | 11.3 | 0.06026 | 2.1 | 0.18264 | 6.3 | 0.11326 | 3.9 | 0.04021 | 1.4 | 0.00045 | 0.0 | 0.00094 | 0.0 | 0.54221 | 18.8 | 0.08951 | 3.1 | 0.00760 | 0.3 | 0.00037 | 0.0 | 1.13587 | 39.4 | 0.00515 | 0.2 | 0.00224 | 0.1 | 0.01968 | 0.7 | - | - |
| 2014 | 0.37613 | 13.1 | 0.29089 | 10.2 | 0.06774 | 2.4 | 0.18468 | 6.5 | 0.11256 | 3.9 | 0.03909 | 1.4 | 0.00039 | 0.0 | 0.03988 | 1.4 | 0.52759 | 18.4 | 0.08314 | 2.9 | 0.00638 | 0.2 | 0.00055 | 0.0 | 1.10585 | 38.6 | 0.00648 | 0.2 | 0.00170 | 0.1 | 0.01850 | 0.6 | - | - |
| 2015 | 0.37379 | 13.0 | 0.30512 | 10.6 | 0.07537 | 2.6 | 0.18968 | 6.6 | 0.11737 | 4.1 | 0.04020 | 1.4 | 0.00248 | 0.1 | 0.04008 | 1.4 | 0.53616 | 18.6 | 0.08033 | 2.8 | 0.00810 | 0.3 | 0.00044 | 0.0 | 1.08583 | 37.7 | 0.00474 | 0.2 | 0.00184 | 0.1 | 0.01974 | 0.7 | - | - |
| 2016 | 0.37423 | 12.8 | 0.30376 | 10.4 | 0.07989 | 2.7 | 0.19321 | 6.6 | 0.11988 | 4.1 | 0.03830 | 1.3 | 0.00266 | 0.1 | 0.04045 | 1.4 | 0.55715 | 19.1 | 0.08061 | 2.8 | 0.00851 | 0.3 | 0.00055 | 0.0 | 1.08601 | 37.3 | 0.00490 | 0.2 | 0.00205 | 0.1 | 0.02058 | 0.7 | - | - |
| 2017 | 0.37854 | 12.9 | 0.31611 | 10.7 | 0.08448 | 2.9 | 0.19673 | 6.7 | 0.12378 | 4.2 | 0.03798 | 1.3 | 0.00290 | 0.1 | 0.04072 | 1.4 | 0.57944 | 19.7 | 0.08055 | 2.7 | 0.00931 | 0.3 | 0.00047 | 0.0 | 1.06189 | 36.1 | 0.00512 | 0.2 | 0.00216 | 0.1 | 0.02131 | 0.7 | - | - |
| 2018 | 0.37480 | 12.6 | 0.33165 | 11.2 | 0.09089 | 3.1 | 0.19563 | 6.6 | 0.12620 | 4.2 | 0.03583 | 1.2 | 0.00344 | 0.1 | 0.04272 | 1.4 | 0.59325 | 20.0 | 0.08212 | 2.8 | 0.00970 | 0.3 | 0.00052 | 0.0 | 1.05534 | 35.5 | 0.00537 | 0.2 | 0.00233 | 0.1 | 0.02296 | 0.8 | - | - |
| 2019 | 0.39699 | 13.2 | 0.34822 | 11.5 | 0.09394 | 3.1 | 0.19335 | 6.4 | 0.12879 | 4.3 | 0.03478 | 1.2 | 0.00309 | 0.1 | 0.04379 | 1.5 | 0.59038 | 19.6 | 0.08175 | 2.7 | 0.00985 | 0.3 | 0.00062 | 0.0 | 1.05865 | 35.1 | 0.00535 | 0.2 | 0.00299 | 0.1 | 0.02245 | 0.7 | - | - |
| 2020 | 0.38506 | 12.8 | 0.38404 | 12.8 | 0.08722 | 2.9 | 0.18050 | 6.0 | 0.12753 | 4.2 | 0.03756 | 1.3 | 0.00354 | 0.1 | 0.04061 | 1.4 | 0.59839 | 19.9 | 0.07763 | 2.6 | 0.00865 | 0.3 | 0.00076 | 0.0 | 1.04585 | 34.8 | 0.00577 | 0.2 | 0.00189 | 0.1 | 0.01719 | 0.6 | - | - |
| 2021 | - | 13.1 | - | 14.0 | - | 3.1 | - | 6.2 | - | 4.1 | - | 1.1 | - | 0.1 | - | 1.3 | - | 20.5 | - | 2.6 | - | 0.4 | - | 0.0 | - | 32.4 | - | 0.2 | - | 0.1 | - | 0.7 | - | - |
