# Supplementary material for: Trends in dental expenditures in Japan with a universal health insurance system
Source: PLoS One. 2023 Oct 5;18(10):e0292547. doi: 10.1371/journal.pone.0292547 (PMC10553203; doi:10.1371/journal.pone.0292547)
Supplement: S2 Table — (DOCX) [file pone.0292547.s002.docx]

**S2 Table. Amount and proportion of services per year for people aged 0-14 years**

| **Year** | **Initial- and repeat-consultation fee (A)** | | **Medical management (B)** | | **At-home treatment (C)** | | **Tests (D)** | | **Diagnostic imaging (E)** | | **Drug administration (F)** | | **Injection (G)** | | **Rehabilitation (H)** | | **Treatment (I)** | | **Surgery (J)** | | **Anaesthesia (K)** | | **Radiotherapy (L)** | | **Crown restoration and prosthesis (M)** | | **Orthodontic treatment (N)** | | **Pathological diagnosis (O)** | | **Hospitalisation fee** | | **Others** | |
| --- | --- | --- | --- | --- | --- | --- | --- | --- | --- | --- | --- | --- | --- | --- | --- | --- | --- | --- | --- | --- | --- | --- | --- | --- | --- | --- | --- | --- | --- | --- | --- | --- | --- | --- |
|  | **Amount** | **%** | **Amount** | **%** | **Amount** | **%** | **Amount** | **%** | **Amount** | **%** | **Amount** | **%** | **Amount** | **%** | **Amount** | **%** | **Amount** | **%** | **Amount** | **%** | **Amount** | **%** | **Amount** | **%** | **Amount** | **%** | **Amount** | **%** | **Amount** | **%** | **Amount** | **%** | **Amount** | **%** |
|  | **(1 trillion yen [≈ 10 billion US dollars])** |  | **(1 trillion yen [≈ 10 billion US dollars])** |  | **(1 trillion yen [≈ 10 billion US dollars])** |  | **(1 trillion yen [≈ 10 billion US dollars])** |  | **(1 trillion yen [≈ 10 billion US dollars])** |  | **(1 trillion yen [≈ 10 billion US dollars])** |  | **(1 trillion yen [≈ 10 billion US dollars])** |  | **(1 trillion yen [≈ 10 billion US dollars])** |  | **(1 trillion yen [≈ 10 billion US dollars])** |  | **(1 trillion yen [≈ 10 billion US dollars])** |  | **(1 trillion yen [≈ 10 billion US dollars])** |  | **(1 trillion yen [≈ 10 billion US dollars])** |  | **(1 trillion yen [≈ 10 billion US dollars])** |  | **(1 trillion yen [≈ 10 billion US dollars])** |  | **(1 trillion yen [≈ 10 billion US dollars])** |  | **(1 trillion yen [≈ 10 billion US dollars])** |  | **(1 trillion yen [≈ 10 billion US dollars])** |  |
| 1996 | 0.04223 | 20.5 | 0.00674 | 3.3 | 0.00000 | 0.0 | 0.00216 | 1.1 | 0.00557 | 2.7 | 0.00229 | 1.1 | 0.00005 | 0.0 | 0.00004 | 0.0 | 0.05491 | 26.7 | 0.00786 | 3.8 | 0.00078 | 0.4 | 0.00000 | 0.0 | 0.08203 | 39.9 | 0.00071 | 0.3 | - | - | 0.00047 | 0.2 | 0.00000 | 0.0 |
| 1997 | 0.03827 | 21.2 | 0.00835 | 4.6 | 0.00013 | 0.1 | 0.00265 | 1.5 | 0.00579 | 3.2 | 0.00230 | 1.3 | 0.00004 | 0.0 | 0.00006 | 0.0 | 0.04775 | 26.4 | 0.00726 | 4.0 | 0.00071 | 0.4 | 0.00000 | 0.0 | 0.06606 | 36.6 | 0.00065 | 0.4 | - | - | 0.00053 | 0.3 | 0.00000 | 0.0 |
| 1998 | 0.04489 | 22.2 | 0.00993 | 4.9 | 0.00000 | 0.0 | 0.00266 | 1.3 | 0.00546 | 2.7 | 0.00258 | 1.3 | 0.00010 | 0.0 | 0.00003 | 0.0 | 0.05210 | 25.7 | 0.00785 | 3.9 | 0.00083 | 0.4 | 0.00000 | 0.0 | 0.07478 | 36.9 | 0.00051 | 0.3 | - | - | 0.00091 | 0.4 | 0.00000 | 0.0 |
| 1999 | 0.04648 | 22.6 | 0.01214 | 5.9 | 0.00000 | 0.0 | 0.00414 | 2.0 | 0.00690 | 3.4 | 0.00231 | 1.1 | 0.00004 | 0.0 | 0.00004 | 0.0 | 0.05345 | 26.0 | 0.00743 | 3.6 | 0.00107 | 0.5 | 0.00000 | 0.0 | 0.07071 | 34.4 | 0.00029 | 0.1 | - | - | 0.00052 | 0.3 | 0.00000 | 0.0 |
| 2000 | 0.04830 | 22.8 | 0.01241 | 5.9 | 0.00012 | 0.1 | 0.00334 | 1.6 | 0.00660 | 3.1 | 0.00280 | 1.3 | 0.00012 | 0.1 | 0.00033 | 0.2 | 0.02523 | 11.9 | 0.00931 | 4.4 | 0.00209 | 1.0 | 0.00000 | 0.0 | 0.09745 | 46.1 | 0.00055 | 0.3 | - | - | 0.00288 | 1.4 | 0.00000 | 0.0 |
| 2001 | 0.04696 | 23.3 | 0.01130 | 5.6 | 0.00000 | 0.0 | 0.00319 | 1.6 | 0.00564 | 2.8 | 0.00232 | 1.1 | 0.00005 | 0.0 | 0.00004 | 0.0 | 0.02372 | 11.7 | 0.00796 | 3.9 | 0.00056 | 0.3 | 0.00000 | 0.0 | 0.09927 | 49.2 | 0.00039 | 0.2 | - | - | 0.00046 | 0.2 | 0.00000 | 0.0 |
| 2002 | 0.05623 | 26.4 | 0.01529 | 7.2 | 0.00000 | 0.0 | 0.00448 | 2.1 | 0.00573 | 2.7 | 0.00191 | 0.9 | 0.00003 | 0.0 | 0.00004 | 0.0 | 0.02496 | 11.7 | 0.00620 | 2.9 | 0.00064 | 0.3 | 0.00000 | 0.0 | 0.09676 | 45.4 | 0.00030 | 0.1 | - | - | 0.00067 | 0.3 | 0.00000 | 0.0 |
| 2003 | 0.05384 | 26.9 | 0.01561 | 7.8 | 0.00000 | 0.0 | 0.00513 | 2.6 | 0.00627 | 3.1 | 0.00190 | 0.9 | 0.00003 | 0.0 | 0.00004 | 0.0 | 0.02652 | 13.2 | 0.00728 | 3.6 | 0.00100 | 0.5 | 0.00000 | 0.0 | 0.08164 | 40.8 | 0.00020 | 0.1 | - | - | 0.00074 | 0.4 | 0.00000 | 0.0 |
| 2004 | 0.05204 | 26.6 | 0.01681 | 8.6 | 0.00000 | 0.0 | 0.00392 | 2.0 | 0.00615 | 3.1 | 0.00226 | 1.2 | 0.00003 | 0.0 | 0.00018 | 0.1 | 0.02758 | 14.1 | 0.00596 | 3.1 | 0.00071 | 0.4 | 0.00000 | 0.0 | 0.07822 | 40.1 | 0.00078 | 0.4 | - | - | 0.00065 | 0.3 | 0.00000 | 0.0 |
| 2005 | 0.05351 | 25.7 | 0.01860 | 8.9 | 0.00000 | 0.0 | 0.00518 | 2.5 | 0.00633 | 3.0 | 0.00212 | 1.0 | 0.00003 | 0.0 | 0.00006 | 0.0 | 0.02684 | 12.9 | 0.00592 | 2.8 | 0.00070 | 0.3 | 0.00000 | 0.0 | 0.08694 | 41.8 | 0.00091 | 0.4 | - | - | 0.00074 | 0.4 | 0.00000 | 0.0 |
| 2006 | 0.04229 | 22.0 | 0.02851 | 14.9 | 0.00000 | 0.0 | 0.00549 | 2.9 | 0.00689 | 3.6 | 0.00192 | 1.0 | 0.00002 | 0.0 | 0.00002 | 0.0 | 0.02505 | 13.1 | 0.00748 | 3.9 | 0.00035 | 0.2 | 0.00000 | 0.0 | 0.07294 | 38.0 | 0.00041 | 0.2 | - | - | 0.00046 | 0.2 | 0.00000 | 0.0 |
| 2007 | 0.04375 | 21.5 | 0.02873 | 14.1 | 0.00014 | 0.1 | 0.00589 | 2.9 | 0.00732 | 3.6 | 0.00169 | 0.8 | 0.00002 | 0.0 | 0.00002 | 0.0 | 0.02639 | 13.0 | 0.00565 | 2.8 | 0.00038 | 0.2 | 0.00000 | 0.0 | 0.08228 | 40.5 | 0.00018 | 0.1 | - | - | 0.00061 | 0.3 | 0.00000 | 0.0 |
| 2008 | 0.04181 | 20.5 | 0.03367 | 16.5 | 0.00000 | 0.0 | 0.00666 | 3.3 | 0.00597 | 2.9 | 0.00223 | 1.1 | 0.00002 | 0.0 | 0.00005 | 0.0 | 0.02895 | 14.2 | 0.00636 | 3.1 | 0.00088 | 0.4 | 0.00000 | 0.0 | 0.07644 | 37.4 | 0.00046 | 0.2 | 0.00002 | 0.0 | 0.00070 | 0.3 | - | - |
| 2009 | 0.03930 | 19.0 | 0.03525 | 17.0 | 0.00000 | 0.0 | 0.00983 | 4.7 | 0.00595 | 2.9 | 0.00174 | 0.8 | 0.00006 | 0.0 | 0.00018 | 0.1 | 0.02815 | 13.6 | 0.00732 | 3.5 | 0.00106 | 0.5 | 0.00000 | 0.0 | 0.07481 | 36.1 | 0.00096 | 0.5 | 0.00009 | 0.0 | 0.00262 | 1.3 | - | - |
| 2010 | 0.04847 | 22.1 | 0.03529 | 16.1 | 0.00000 | 0.0 | 0.00739 | 3.4 | 0.00624 | 2.8 | 0.00189 | 0.9 | 0.00003 | 0.0 | 0.00036 | 0.2 | 0.03153 | 14.3 | 0.00767 | 3.5 | 0.00077 | 0.3 | 0.00000 | 0.0 | 0.07839 | 35.7 | 0.00020 | 0.1 | 0.00012 | 0.1 | 0.00140 | 0.6 | - | - |
| 2011 | 0.05035 | 22.4 | 0.03859 | 17.2 | 0.00003 | 0.0 | 0.00825 | 3.7 | 0.00764 | 3.4 | 0.00213 | 0.9 | 0.00003 | 0.0 | 0.00017 | 0.1 | 0.03585 | 16.0 | 0.00773 | 3.4 | 0.00153 | 0.7 | 0.00000 | 0.0 | 0.06981 | 31.1 | 0.00089 | 0.4 | 0.00006 | 0.0 | 0.00159 | 0.7 | - | - |
| 2012 | 0.04974 | 21.6 | 0.03369 | 14.7 | 0.00005 | 0.0 | 0.00754 | 3.3 | 0.00776 | 3.4 | 0.00178 | 0.8 | 0.00001 | 0.0 | 0.00074 | 0.3 | 0.04200 | 18.3 | 0.00645 | 2.8 | 0.00072 | 0.3 | 0.00000 | 0.0 | 0.07776 | 33.8 | 0.00080 | 0.3 | 0.00006 | 0.0 | 0.00076 | 0.3 | - | - |
| 2013 | 0.04935 | 21.5 | 0.03432 | 15.0 | 0.00000 | 0.0 | 0.00833 | 3.6 | 0.00769 | 3.4 | 0.00205 | 0.9 | 0.00000 | 0.0 | 0.00004 | 0.0 | 0.04691 | 20.4 | 0.00667 | 2.9 | 0.00060 | 0.3 | 0.00000 | 0.0 | 0.07249 | 31.6 | 0.00019 | 0.1 | 0.00016 | 0.1 | 0.00069 | 0.3 | - | - |
| 2014 | 0.05184 | 22.7 | 0.03388 | 14.9 | 0.00000 | 0.0 | 0.00857 | 3.8 | 0.00752 | 3.3 | 0.00187 | 0.8 | 0.00001 | 0.0 | 0.00018 | 0.1 | 0.04439 | 19.5 | 0.00639 | 2.8 | 0.00083 | 0.4 | 0.00000 | 0.0 | 0.07039 | 30.9 | 0.00109 | 0.5 | 0.00006 | 0.0 | 0.00099 | 0.4 | - | - |
| 2015 | 0.05191 | 22.5 | 0.03531 | 15.3 | 0.00011 | 0.0 | 0.00920 | 4.0 | 0.00773 | 3.4 | 0.00177 | 0.8 | 0.00002 | 0.0 | 0.00017 | 0.1 | 0.04784 | 20.8 | 0.00606 | 2.6 | 0.00099 | 0.4 | 0.00000 | 0.0 | 0.06736 | 29.2 | 0.00094 | 0.4 | 0.00006 | 0.0 | 0.00098 | 0.4 | - | - |
| 2016 | 0.05263 | 22.0 | 0.03674 | 15.3 | 0.00013 | 0.1 | 0.01128 | 4.7 | 0.00819 | 3.4 | 0.00170 | 0.7 | 0.00003 | 0.0 | 0.00017 | 0.1 | 0.05302 | 22.2 | 0.00613 | 2.6 | 0.00106 | 0.4 | 0.00000 | 0.0 | 0.06594 | 27.5 | 0.00098 | 0.4 | 0.00007 | 0.0 | 0.00128 | 0.5 | - | - |
| 2017 | 0.05283 | 21.6 | 0.04062 | 16.6 | 0.00016 | 0.1 | 0.01191 | 4.9 | 0.00851 | 3.5 | 0.00165 | 0.7 | 0.00002 | 0.0 | 0.00017 | 0.1 | 0.05478 | 22.4 | 0.00603 | 2.5 | 0.00108 | 0.4 | 0.00000 | 0.0 | 0.06424 | 26.3 | 0.00100 | 0.4 | 0.00007 | 0.0 | 0.00105 | 0.4 | - | - |
| 2018 | 0.05305 | 21.2 | 0.04529 | 18.1 | 0.00020 | 0.1 | 0.01221 | 4.9 | 0.00884 | 3.5 | 0.00158 | 0.6 | 0.00002 | 0.0 | 0.00019 | 0.1 | 0.05794 | 23.1 | 0.00584 | 2.3 | 0.00117 | 0.5 | 0.00000 | 0.0 | 0.06200 | 24.7 | 0.00100 | 0.4 | 0.00007 | 0.0 | 0.00115 | 0.5 | - | - |
| 2019 | 0.04888 | 19.2 | 0.04572 | 18.0 | 0.00027 | 0.1 | 0.01537 | 6.0 | 0.01164 | 4.6 | 0.00197 | 0.8 | 0.00004 | 0.0 | 0.00013 | 0.1 | 0.05659 | 22.3 | 0.00670 | 2.6 | 0.00129 | 0.5 | 0.00000 | 0.0 | 0.06137 | 24.2 | 0.00249 | 1.0 | 0.00013 | 0.1 | 0.00141 | 0.6 | - | - |
| 2020 | 0.05167 | 20.6 | 0.05153 | 20.6 | 0.00022 | 0.1 | 0.01263 | 5.0 | 0.00926 | 3.7 | 0.00171 | 0.7 | 0.00001 | 0.0 | 0.00011 | 0.0 | 0.05930 | 23.7 | 0.00572 | 2.3 | 0.00119 | 0.5 | 0.00000 | 0.0 | 0.05507 | 22.0 | 0.00097 | 0.4 | 0.00007 | 0.0 | 0.00093 | 0.4 | - | - |
| 2021 | - | 22.5 | - | 22.6 | - | 0.1 | - | 5.3 | - | 3.4 | - | 0.5 | - | 0.0 | - | 0.1 | - | 23.3 | - | 2.0 | - | 0.5 | - | 0.0 | - | 18.7 | - | 0.4 | - | 0.0 | - | 0.5 | - | - |
