# Supplementary material for: Trends in dental expenditures in Japan with a universal health insurance system
Source: PLoS One. 2023 Oct 5;18(10):e0292547. doi: 10.1371/journal.pone.0292547 (PMC10553203; doi:10.1371/journal.pone.0292547)
Supplement: S3 Table — (DOCX) [file pone.0292547.s003.docx]

**S3 Table. Amount and proportion of services per year for people aged 15-44 years**

| **Year** | **Initial- and repeat-consultation fee (A)** | | **Medical management (B)** | | **At-home treatment (C)** | | **Tests (D)** | | **Diagnostic imaging (E)** | | **Drug administration (F)** | | **Injection (G)** | | **Rehabilitation (H)** | | **Treatment (I)** | | **Surgery (J)** | | **Anaesthesia (K)** | | **Radiotherapy (L)** | | **Crown restoration and prosthesis (M)** | | **Orthodontic treatment (N)** | | **Pathological diagnosis (O)** | | **Hospitalisation fee** | | **Others** | |
| --- | --- | --- | --- | --- | --- | --- | --- | --- | --- | --- | --- | --- | --- | --- | --- | --- | --- | --- | --- | --- | --- | --- | --- | --- | --- | --- | --- | --- | --- | --- | --- | --- | --- | --- |
|  | **Amount** | **%** | **Amount** | **%** | **Amount** | **%** | **Amount** | **%** | **Amount** | **%** | **Amount** | **%** | **Amount** | **%** | **Amount** | **%** | **Amount** | **%** | **Amount** | **%** | **Amount** | **%** | **Amount** | **%** | **Amount** | **%** | **Amount** | **%** | **Amount** | **%** | **Amount** | **%** | **Amount** | **%** |
|  | **(1 trillion yen [≈ 10 billion US dollars])** |  | **(1 trillion yen [≈ 10 billion US dollars])** |  | **(1 trillion yen [≈ 10 billion US dollars])** |  | **(1 trillion yen [≈ 10 billion US dollars])** |  | **(1 trillion yen [≈ 10 billion US dollars])** |  | **(1 trillion yen [≈ 10 billion US dollars])** |  | **(1 trillion yen [≈ 10 billion US dollars])** |  | **(1 trillion yen [≈ 10 billion US dollars])** |  | **(1 trillion yen [≈ 10 billion US dollars])** |  | **(1 trillion yen [≈ 10 billion US dollars])** |  | **(1 trillion yen [≈ 10 billion US dollars])** |  | **(1 trillion yen [≈ 10 billion US dollars])** |  | **(1 trillion yen [≈ 10 billion US dollars])** |  | **(1 trillion yen [≈ 10 billion US dollars])** |  | **(1 trillion yen [≈ 10 billion US dollars])** |  | **(1 trillion yen [≈ 10 billion US dollars])** |  | **(1 trillion yen [≈ 10 billion US dollars])** |  |
| 1996 | 0.09904 | 10.8 | 0.04973 | 5.4 | 0.00091 | 0.1 | 0.05893 | 6.4 | 0.04367 | 4.8 | 0.02109 | 2.3 | 0.00106 | 0.1 | 0.00010 | 0.0 | 0.21393 | 23.3 | 0.03432 | 3.7 | 0.00385 | 0.4 | 0.00000 | 0.0 | 0.38622 | 42.1 | 0.00016 | 0.0 | - | - | 0.00502 | 0.5 | 0.00000 | 0.0 |
| 1997 | 0.09042 | 10.2 | 0.05326 | 6.0 | 0.00005 | 0.0 | 0.05419 | 6.1 | 0.04432 | 5.0 | 0.01971 | 2.2 | 0.00124 | 0.1 | 0.00033 | 0.0 | 0.20160 | 22.8 | 0.03216 | 3.6 | 0.00343 | 0.4 | 0.00007 | 0.0 | 0.37334 | 42.3 | 0.00404 | 0.5 | - | - | 0.00534 | 0.6 | 0.00000 | 0.0 |
| 1998 | 0.09418 | 11.2 | 0.05839 | 7.0 | 0.00015 | 0.0 | 0.05038 | 6.0 | 0.03848 | 4.6 | 0.01842 | 2.2 | 0.00114 | 0.1 | 0.00011 | 0.0 | 0.19741 | 23.5 | 0.03337 | 4.0 | 0.00350 | 0.4 | 0.00001 | 0.0 | 0.33676 | 40.1 | 0.00134 | 0.2 | - | - | 0.00563 | 0.7 | 0.00000 | 0.0 |
| 1999 | 0.08932 | 11.3 | 0.06047 | 7.6 | 0.00029 | 0.0 | 0.05223 | 6.6 | 0.03900 | 4.9 | 0.01765 | 2.2 | 0.00070 | 0.1 | 0.00016 | 0.0 | 0.19082 | 24.1 | 0.03073 | 3.9 | 0.00307 | 0.4 | 0.00000 | 0.0 | 0.30360 | 38.3 | 0.00024 | 0.0 | - | - | 0.00419 | 0.5 | 0.00000 | 0.0 |
| 2000 | 0.09139 | 11.7 | 0.06154 | 7.9 | 0.00054 | 0.1 | 0.05344 | 6.8 | 0.03489 | 4.5 | 0.01770 | 2.3 | 0.00101 | 0.1 | 0.00013 | 0.0 | 0.14792 | 18.9 | 0.03108 | 4.0 | 0.00351 | 0.4 | 0.00001 | 0.0 | 0.32814 | 42.0 | 0.00304 | 0.4 | - | - | 0.00687 | 0.9 | 0.00000 | 0.0 |
| 2001 | 0.09317 | 11.6 | 0.06128 | 7.6 | 0.00033 | 0.0 | 0.05109 | 6.4 | 0.03383 | 4.2 | 0.01737 | 2.2 | 0.00069 | 0.1 | 0.00007 | 0.0 | 0.14496 | 18.1 | 0.03143 | 3.9 | 0.00364 | 0.5 | 0.00001 | 0.0 | 0.35828 | 44.6 | 0.00172 | 0.2 | - | - | 0.00501 | 0.6 | 0.00000 | 0.0 |
| 2002 | 0.11260 | 13.3 | 0.07169 | 8.5 | 0.00002 | 0.0 | 0.05983 | 7.1 | 0.04113 | 4.9 | 0.01675 | 2.0 | 0.00074 | 0.1 | 0.00017 | 0.0 | 0.16258 | 19.2 | 0.03164 | 3.7 | 0.00317 | 0.4 | 0.00002 | 0.0 | 0.34117 | 40.2 | 0.00065 | 0.1 | - | - | 0.00585 | 0.7 | 0.00000 | 0.0 |
| 2003 | 0.10985 | 13.8 | 0.06768 | 8.5 | 0.00047 | 0.1 | 0.05504 | 6.9 | 0.03717 | 4.7 | 0.01697 | 2.1 | 0.00076 | 0.1 | 0.00008 | 0.0 | 0.16306 | 20.4 | 0.03091 | 3.9 | 0.00378 | 0.5 | 0.00002 | 0.0 | 0.30521 | 38.2 | 0.00067 | 0.1 | - | - | 0.00655 | 0.8 | 0.00000 | 0.0 |
| 2004 | 0.10979 | 13.9 | 0.07399 | 9.4 | 0.00148 | 0.2 | 0.05604 | 7.1 | 0.03873 | 4.9 | 0.01684 | 2.1 | 0.00052 | 0.1 | 0.00007 | 0.0 | 0.15244 | 19.4 | 0.03210 | 4.1 | 0.00273 | 0.3 | 0.00003 | 0.0 | 0.29647 | 37.6 | 0.00265 | 0.3 | - | - | 0.00377 | 0.5 | 0.00000 | 0.0 |
| 2005 | 0.11264 | 14.4 | 0.07470 | 9.6 | 0.00091 | 0.1 | 0.05655 | 7.2 | 0.04010 | 5.1 | 0.01744 | 2.2 | 0.00100 | 0.1 | 0.00004 | 0.0 | 0.15889 | 20.4 | 0.02810 | 3.6 | 0.00327 | 0.4 | 0.00001 | 0.0 | 0.28078 | 36.0 | 0.00099 | 0.1 | - | - | 0.00526 | 0.7 | 0.00000 | 0.0 |
| 2006 | 0.08553 | 12.1 | 0.07660 | 10.8 | 0.00000 | 0.0 | 0.05680 | 8.0 | 0.03866 | 5.5 | 0.01560 | 2.2 | 0.00063 | 0.1 | 0.00005 | 0.0 | 0.13508 | 19.1 | 0.02691 | 3.8 | 0.00302 | 0.4 | 0.00001 | 0.0 | 0.26146 | 37.0 | 0.00244 | 0.3 | - | - | 0.00467 | 0.7 | 0.00000 | 0.0 |
| 2007 | 0.08334 | 11.8 | 0.07349 | 10.4 | 0.00222 | 0.3 | 0.05368 | 7.6 | 0.03818 | 5.4 | 0.01378 | 1.9 | 0.00060 | 0.1 | 0.00006 | 0.0 | 0.14545 | 20.5 | 0.02281 | 3.2 | 0.00279 | 0.4 | 0.00001 | 0.0 | 0.26764 | 37.7 | 0.00063 | 0.1 | - | - | 0.00454 | 0.6 | 0.00000 | 0.0 |
| 2008 | 0.08615 | 11.8 | 0.08977 | 12.3 | 0.00017 | 0.0 | 0.05809 | 8.0 | 0.04071 | 5.6 | 0.01372 | 1.9 | 0.00063 | 0.1 | 0.00005 | 0.0 | 0.14297 | 19.6 | 0.02248 | 3.1 | 0.00282 | 0.4 | 0.00005 | 0.0 | 0.26303 | 36.0 | 0.00425 | 0.6 | 0.00021 | 0.0 | 0.00547 | 0.7 | - | - |
| 2009 | 0.08646 | 11.9 | 0.09151 | 12.6 | 0.00107 | 0.1 | 0.05681 | 7.8 | 0.03617 | 5.0 | 0.01338 | 1.8 | 0.00065 | 0.1 | 0.00007 | 0.0 | 0.14119 | 19.5 | 0.02219 | 3.1 | 0.00303 | 0.4 | 0.00001 | 0.0 | 0.26171 | 36.1 | 0.00403 | 0.6 | 0.00045 | 0.1 | 0.00640 | 0.9 | - | - |
| 2010 | 0.09651 | 13.3 | 0.08761 | 12.1 | 0.00694 | 1.0 | 0.05719 | 7.9 | 0.03936 | 5.4 | 0.01400 | 1.9 | 0.00069 | 0.1 | 0.00006 | 0.0 | 0.14549 | 20.0 | 0.02831 | 3.9 | 0.00321 | 0.4 | 0.00008 | 0.0 | 0.23555 | 32.5 | 0.00344 | 0.5 | 0.00041 | 0.1 | 0.00689 | 0.9 | - | - |
| 2011 | 0.09957 | 13.4 | 0.09215 | 12.4 | 0.00099 | 0.1 | 0.06000 | 8.1 | 0.04037 | 5.4 | 0.01318 | 1.8 | 0.00032 | 0.0 | 0.00005 | 0.0 | 0.14342 | 19.3 | 0.02437 | 3.3 | 0.00311 | 0.4 | 0.00001 | 0.0 | 0.25705 | 34.5 | 0.00416 | 0.6 | 0.00027 | 0.0 | 0.00521 | 0.7 | - | - |
| 2012 | 0.09734 | 13.1 | 0.07570 | 10.2 | 0.00269 | 0.4 | 0.05866 | 7.9 | 0.04308 | 5.8 | 0.01229 | 1.7 | 0.00010 | 0.0 | 0.00007 | 0.0 | 0.16038 | 21.6 | 0.02429 | 3.3 | 0.00353 | 0.5 | 0.00001 | 0.0 | 0.25655 | 34.6 | 0.00120 | 0.2 | 0.00034 | 0.0 | 0.00547 | 0.7 | - | - |
| 2013 | 0.09664 | 13.2 | 0.07648 | 10.5 | 0.00135 | 0.2 | 0.05937 | 8.1 | 0.04553 | 6.2 | 0.01189 | 1.6 | 0.00011 | 0.0 | 0.00003 | 0.0 | 0.15769 | 21.6 | 0.02811 | 3.8 | 0.00379 | 0.5 | 0.00004 | 0.0 | 0.23744 | 32.5 | 0.00503 | 0.7 | 0.00074 | 0.1 | 0.00622 | 0.9 | - | - |
| 2014 | 0.10236 | 14.2 | 0.07659 | 10.6 | 0.00110 | 0.2 | 0.06026 | 8.4 | 0.04299 | 6.0 | 0.01113 | 1.5 | 0.00010 | 0.0 | 0.00051 | 0.1 | 0.15523 | 21.5 | 0.02526 | 3.5 | 0.00290 | 0.4 | 0.00003 | 0.0 | 0.23172 | 32.1 | 0.00534 | 0.7 | 0.00039 | 0.1 | 0.00549 | 0.8 | - | - |
| 2015 | 0.09891 | 13.8 | 0.07662 | 10.7 | 0.00189 | 0.3 | 0.05969 | 8.3 | 0.04427 | 6.2 | 0.01162 | 1.6 | 0.00048 | 0.1 | 0.00052 | 0.1 | 0.15250 | 21.3 | 0.02440 | 3.4 | 0.00370 | 0.5 | 0.00002 | 0.0 | 0.23246 | 32.4 | 0.00352 | 0.5 | 0.00039 | 0.1 | 0.00579 | 0.8 | - | - |
| 2016 | 0.09790 | 13.7 | 0.07525 | 10.5 | 0.00201 | 0.3 | 0.05965 | 8.4 | 0.04493 | 6.3 | 0.01096 | 1.5 | 0.00040 | 0.1 | 0.00052 | 0.1 | 0.15536 | 21.8 | 0.02470 | 3.5 | 0.00384 | 0.5 | 0.00003 | 0.0 | 0.22845 | 32.0 | 0.00369 | 0.5 | 0.00045 | 0.1 | 0.00582 | 0.8 | - | - |
| 2017 | 0.09814 | 13.8 | 0.07698 | 10.8 | 0.00210 | 0.3 | 0.06031 | 8.5 | 0.04624 | 6.5 | 0.01073 | 1.5 | 0.00047 | 0.1 | 0.00052 | 0.1 | 0.15750 | 22.1 | 0.02509 | 3.5 | 0.00428 | 0.6 | 0.00003 | 0.0 | 0.21856 | 30.7 | 0.00391 | 0.5 | 0.00045 | 0.1 | 0.00626 | 0.9 | - | - |
| 2018 | 0.09569 | 13.6 | 0.07911 | 11.3 | 0.00221 | 0.3 | 0.05857 | 8.4 | 0.04647 | 6.6 | 0.00981 | 1.4 | 0.00048 | 0.1 | 0.00053 | 0.1 | 0.15486 | 22.1 | 0.02532 | 3.6 | 0.00444 | 0.6 | 0.00002 | 0.0 | 0.21239 | 30.3 | 0.00422 | 0.6 | 0.00050 | 0.1 | 0.00661 | 0.9 | - | - |
| 2019 | 0.09493 | 13.6 | 0.08220 | 11.8 | 0.00267 | 0.4 | 0.05874 | 8.4 | 0.04472 | 6.4 | 0.00939 | 1.3 | 0.00049 | 0.1 | 0.00088 | 0.1 | 0.15999 | 23.0 | 0.02428 | 3.5 | 0.00406 | 0.6 | 0.00003 | 0.0 | 0.20481 | 29.4 | 0.00253 | 0.4 | 0.00066 | 0.1 | 0.00622 | 0.9 | - | - |
| 2020 | 0.09917 | 13.8 | 0.08351 | 11.6 | 0.00168 | 0.2 | 0.05404 | 7.5 | 0.04703 | 6.5 | 0.01002 | 1.4 | 0.00034 | 0.0 | 0.00050 | 0.1 | 0.15821 | 22.0 | 0.02326 | 3.2 | 0.00324 | 0.5 | 0.00005 | 0.0 | 0.22928 | 31.9 | 0.00438 | 0.6 | 0.00036 | 0.1 | 0.00383 | 0.5 | - | - |
| 2021 | - | 14.1 | - | 13.2 | - | 0.3 | - | 8.0 | - | 6.5 | - | 1.2 | - | 0.1 | - | 0.1 | - | 22.5 | - | 3.7 | - | 0.7 | - | 0.0 | - | 27.8 | - | 0.8 | - | 0.1 | - | 1.0 | - | - |
