# Supplementary material for: Trends in dental expenditures in Japan with a universal health insurance system
Source: PLoS One. 2023 Oct 5;18(10):e0292547. doi: 10.1371/journal.pone.0292547 (PMC10553203; doi:10.1371/journal.pone.0292547)
Supplement: S4 Table — (DOCX) [file pone.0292547.s004.docx]

**S4 Table. Amount and proportion of services per year for people aged 45-64 years**

| **Year** | **Initial- and repeat-consultation fee (A)** | | **Medical management (B)** | | **At-home treatment (C)** | | **Tests (D)** | | **Diagnostic imaging (E)** | | **Drug administration (F)** | | **Injection (G)** | | **Rehabilitation (H)** | | **Treatment (I)** | | **Surgery (J)** | | **Anaesthesia (K)** | | **Radiotherapy (L)** | | **Crown restoration and prosthesis (M)** | | **Orthodontic treatment (N)** | | **Pathological diagnosis (O)** | | **Hospitalisation fee** | | **Others** | |
| --- | --- | --- | --- | --- | --- | --- | --- | --- | --- | --- | --- | --- | --- | --- | --- | --- | --- | --- | --- | --- | --- | --- | --- | --- | --- | --- | --- | --- | --- | --- | --- | --- | --- | --- |
|  | **Amount** | **%** | **Amount** | **%** | **Amount** | **%** | **Amount** | **%** | **Amount** | **%** | **Amount** | **%** | **Amount** | **%** | **Amount** | **%** | **Amount** | **%** | **Amount** | **%** | **Amount** | **%** | **Amount** | **%** | **Amount** | **%** | **Amount** | **%** | **Amount** | **%** | **Amount** | **%** | **Amount** | **%** |
|  | **(1 trillion yen [≈ 10 billion US dollars])** |  | **(1 trillion yen [≈ 10 billion US dollars])** |  | **(1 trillion yen [≈ 10 billion US dollars])** |  | **(1 trillion yen [≈ 10 billion US dollars])** |  | **(1 trillion yen [≈ 10 billion US dollars])** |  | **(1 trillion yen [≈ 10 billion US dollars])** |  | **(1 trillion yen [≈ 10 billion US dollars])** |  | **(1 trillion yen [≈ 10 billion US dollars])** |  | **(1 trillion yen [≈ 10 billion US dollars])** |  | **(1 trillion yen [≈ 10 billion US dollars])** |  | **(1 trillion yen [≈ 10 billion US dollars])** |  | **(1 trillion yen [≈ 10 billion US dollars])** |  | **(1 trillion yen [≈ 10 billion US dollars])** |  | **(1 trillion yen [≈ 10 billion US dollars])** |  | **(1 trillion yen [≈ 10 billion US dollars])** |  | **(1 trillion yen [≈ 10 billion US dollars])** |  | **(1 trillion yen [≈ 10 billion US dollars])** |  |
| 1996 | 0.09688 | 9.6 | 0.04656 | 4.6 | 0.00059 | 0.1 | 0.05588 | 5.6 | 0.03571 | 3.6 | 0.01869 | 1.9 | 0.00157 | 0.2 | 0.00003 | 0.0 | 0.18096 | 18.0 | 0.04021 | 4.0 | 0.00236 | 0.2 | 0.00022 | 0.0 | 0.51961 | 51.7 | 0.00000 | 0.0 | - | - | 0.00511 | 0.5 | 0.00000 | 0.0 |
| 1997 | 0.09669 | 9.8 | 0.05561 | 5.6 | 0.00115 | 0.1 | 0.05387 | 5.4 | 0.03713 | 3.7 | 0.01995 | 2.0 | 0.00093 | 0.1 | 0.00020 | 0.0 | 0.18517 | 18.7 | 0.03780 | 3.8 | 0.00220 | 0.2 | 0.00015 | 0.0 | 0.49716 | 50.2 | 0.00000 | 0.0 | - | - | 0.00317 | 0.3 | 0.00000 | 0.0 |
| 1998 | 0.09623 | 10.1 | 0.05783 | 6.1 | 0.00047 | 0.0 | 0.04927 | 5.2 | 0.03112 | 3.3 | 0.01782 | 1.9 | 0.00095 | 0.1 | 0.00018 | 0.0 | 0.17401 | 18.3 | 0.03813 | 4.0 | 0.00177 | 0.2 | 0.00007 | 0.0 | 0.47795 | 50.3 | 0.00000 | 0.0 | - | - | 0.00466 | 0.5 | 0.00001 | 0.0 |
| 1999 | 0.09624 | 10.0 | 0.06445 | 6.7 | 0.00135 | 0.1 | 0.05216 | 5.4 | 0.03259 | 3.4 | 0.01797 | 1.9 | 0.00103 | 0.1 | 0.00002 | 0.0 | 0.17731 | 18.5 | 0.03644 | 3.8 | 0.00179 | 0.2 | 0.00009 | 0.0 | 0.47375 | 49.4 | 0.00000 | 0.0 | - | - | 0.00470 | 0.5 | 0.00000 | 0.0 |
| 2000 | 0.09985 | 10.3 | 0.06769 | 7.0 | 0.00084 | 0.1 | 0.05788 | 6.0 | 0.03259 | 3.4 | 0.01810 | 1.9 | 0.00083 | 0.1 | 0.00018 | 0.0 | 0.15914 | 16.4 | 0.03631 | 3.7 | 0.00212 | 0.2 | 0.00016 | 0.0 | 0.49168 | 50.6 | 0.00000 | 0.0 | - | - | 0.00487 | 0.5 | 0.00000 | 0.0 |
| 2001 | 0.10243 | 10.5 | 0.06963 | 7.1 | 0.00007 | 0.0 | 0.05584 | 5.7 | 0.02900 | 3.0 | 0.01866 | 1.9 | 0.00067 | 0.1 | 0.00002 | 0.0 | 0.15753 | 16.1 | 0.03677 | 3.8 | 0.00171 | 0.2 | 0.00005 | 0.0 | 0.50223 | 51.4 | 0.00000 | 0.0 | - | - | 0.00318 | 0.3 | 0.00000 | 0.0 |
| 2002 | 0.10909 | 11.7 | 0.06892 | 7.4 | 0.00021 | 0.0 | 0.05297 | 5.7 | 0.03205 | 3.4 | 0.01658 | 1.8 | 0.00072 | 0.1 | 0.00025 | 0.0 | 0.15190 | 16.3 | 0.03919 | 4.2 | 0.00172 | 0.2 | 0.00010 | 0.0 | 0.45219 | 48.6 | 0.00000 | 0.0 | - | - | 0.00500 | 0.5 | 0.00000 | 0.0 |
| 2003 | 0.10416 | 11.1 | 0.06520 | 6.9 | 0.00139 | 0.1 | 0.05234 | 5.6 | 0.02941 | 3.1 | 0.01650 | 1.8 | 0.00097 | 0.1 | 0.00020 | 0.0 | 0.14962 | 15.9 | 0.03566 | 3.8 | 0.00186 | 0.2 | 0.00010 | 0.0 | 0.47827 | 50.8 | 0.00000 | 0.0 | - | - | 0.00579 | 0.6 | 0.00000 | 0.0 |
| 2004 | 0.11701 | 12.9 | 0.07512 | 8.3 | 0.00171 | 0.2 | 0.05349 | 5.9 | 0.03127 | 3.4 | 0.01812 | 2.0 | 0.00085 | 0.1 | 0.00004 | 0.0 | 0.14497 | 16.0 | 0.03943 | 4.3 | 0.00213 | 0.2 | 0.00008 | 0.0 | 0.41944 | 46.3 | 0.00000 | 0.0 | - | - | 0.00305 | 0.3 | 0.00000 | 0.0 |
| 2005 | 0.11886 | 13.0 | 0.07433 | 8.1 | 0.00112 | 0.1 | 0.05486 | 6.0 | 0.03146 | 3.4 | 0.01745 | 1.9 | 0.00108 | 0.1 | 0.00012 | 0.0 | 0.15286 | 16.7 | 0.03211 | 3.5 | 0.00174 | 0.2 | 0.00016 | 0.0 | 0.42260 | 46.3 | 0.00000 | 0.0 | - | - | 0.00406 | 0.4 | 0.00000 | 0.0 |
| 2006 | 0.09543 | 10.6 | 0.09130 | 10.1 | 0.00237 | 0.3 | 0.06059 | 6.7 | 0.03075 | 3.4 | 0.01679 | 1.9 | 0.00086 | 0.1 | 0.00004 | 0.0 | 0.15121 | 16.8 | 0.02951 | 3.3 | 0.00170 | 0.2 | 0.00011 | 0.0 | 0.41747 | 46.3 | 0.00000 | 0.0 | - | - | 0.00415 | 0.5 | 0.00000 | 0.0 |
| 2007 | 0.09220 | 10.5 | 0.08314 | 9.4 | 0.00183 | 0.2 | 0.05719 | 6.5 | 0.03047 | 3.5 | 0.01645 | 1.9 | 0.00071 | 0.1 | 0.00005 | 0.0 | 0.15916 | 18.1 | 0.02833 | 3.2 | 0.00152 | 0.2 | 0.00010 | 0.0 | 0.40548 | 46.0 | 0.00000 | 0.0 | - | - | 0.00433 | 0.5 | 0.00000 | 0.0 |
| 2008 | 0.09388 | 11.0 | 0.10506 | 12.3 | 0.00097 | 0.1 | 0.06045 | 7.1 | 0.03015 | 3.5 | 0.01496 | 1.7 | 0.00069 | 0.1 | 0.00010 | 0.0 | 0.14943 | 17.5 | 0.02562 | 3.0 | 0.00193 | 0.2 | 0.00011 | 0.0 | 0.36640 | 42.8 | 0.00005 | 0.0 | 0.00044 | 0.1 | 0.00523 | 0.6 | - | - |
| 2009 | 0.09304 | 11.0 | 0.10785 | 12.8 | 0.00658 | 0.8 | 0.05918 | 7.0 | 0.03349 | 4.0 | 0.01626 | 1.9 | 0.00058 | 0.1 | 0.00009 | 0.0 | 0.14770 | 17.5 | 0.02829 | 3.4 | 0.00197 | 0.2 | 0.00006 | 0.0 | 0.34190 | 40.6 | 0.00008 | 0.0 | 0.00060 | 0.1 | 0.00536 | 0.6 | - | - |
| 2010 | 0.10289 | 12.1 | 0.10696 | 12.5 | 0.00670 | 0.8 | 0.05985 | 7.0 | 0.03180 | 3.7 | 0.01479 | 1.7 | 0.00060 | 0.1 | 0.00022 | 0.0 | 0.14913 | 17.5 | 0.02534 | 3.0 | 0.00180 | 0.2 | 0.00012 | 0.0 | 0.34740 | 40.7 | 0.00001 | 0.0 | 0.00078 | 0.1 | 0.00541 | 0.6 | - | - |
| 2011 | 0.10228 | 11.7 | 0.11146 | 12.8 | 0.00456 | 0.5 | 0.06369 | 7.3 | 0.03143 | 3.6 | 0.01388 | 1.6 | 0.00047 | 0.1 | 0.00012 | 0.0 | 0.15502 | 17.8 | 0.02673 | 3.1 | 0.00152 | 0.2 | 0.00006 | 0.0 | 0.35675 | 40.9 | 0.00012 | 0.0 | 0.00052 | 0.1 | 0.00377 | 0.4 | - | - |
| 2012 | 0.10276 | 11.9 | 0.09304 | 10.8 | 0.00489 | 0.6 | 0.05995 | 7.0 | 0.03308 | 3.8 | 0.01317 | 1.5 | 0.00015 | 0.0 | 0.00009 | 0.0 | 0.16880 | 19.6 | 0.02368 | 2.7 | 0.00166 | 0.2 | 0.00015 | 0.0 | 0.35510 | 41.2 | 0.00014 | 0.0 | 0.00063 | 0.1 | 0.00492 | 0.6 | - | - |
| 2013 | 0.09911 | 11.8 | 0.09249 | 11.1 | 0.00339 | 0.4 | 0.05984 | 7.2 | 0.03272 | 3.9 | 0.01226 | 1.5 | 0.00007 | 0.0 | 0.00006 | 0.0 | 0.16845 | 20.1 | 0.02563 | 3.1 | 0.00165 | 0.2 | 0.00008 | 0.0 | 0.33617 | 40.2 | 0.00011 | 0.0 | 0.00054 | 0.1 | 0.00409 | 0.5 | - | - |
| 2014 | 0.10322 | 12.7 | 0.08389 | 10.3 | 0.00360 | 0.4 | 0.05895 | 7.2 | 0.03314 | 4.1 | 0.01200 | 1.5 | 0.00009 | 0.0 | 0.00734 | 0.9 | 0.16375 | 20.1 | 0.02359 | 2.9 | 0.00143 | 0.2 | 0.00009 | 0.0 | 0.31747 | 39.0 | 0.00023 | 0.0 | 0.00053 | 0.1 | 0.00391 | 0.5 | - | - |
| 2015 | 0.09964 | 12.3 | 0.08581 | 10.6 | 0.00415 | 0.5 | 0.05971 | 7.4 | 0.03355 | 4.2 | 0.01191 | 1.5 | 0.00063 | 0.1 | 0.00693 | 0.9 | 0.16224 | 20.1 | 0.02167 | 2.7 | 0.00177 | 0.2 | 0.00011 | 0.0 | 0.31420 | 38.9 | 0.00019 | 0.0 | 0.00057 | 0.1 | 0.00437 | 0.5 | - | - |
| 2016 | 0.09955 | 12.3 | 0.08460 | 10.4 | 0.00440 | 0.5 | 0.06007 | 7.4 | 0.03428 | 4.2 | 0.01123 | 1.4 | 0.00075 | 0.1 | 0.00657 | 0.8 | 0.16734 | 20.6 | 0.02133 | 2.6 | 0.00192 | 0.2 | 0.00016 | 0.0 | 0.31370 | 38.7 | 0.00021 | 0.0 | 0.00061 | 0.1 | 0.00428 | 0.5 | - | - |
| 2017 | 0.10146 | 12.4 | 0.08758 | 10.7 | 0.00475 | 0.6 | 0.06153 | 7.5 | 0.03567 | 4.4 | 0.01120 | 1.4 | 0.00075 | 0.1 | 0.00633 | 0.8 | 0.17542 | 21.4 | 0.02122 | 2.6 | 0.00210 | 0.3 | 0.00012 | 0.0 | 0.30546 | 37.3 | 0.00022 | 0.0 | 0.00066 | 0.1 | 0.00440 | 0.5 | - | - |
| 2018 | 0.10115 | 12.2 | 0.09154 | 11.1 | 0.00498 | 0.6 | 0.06139 | 7.4 | 0.03669 | 4.4 | 0.01059 | 1.3 | 0.00094 | 0.1 | 0.00628 | 0.8 | 0.17978 | 21.8 | 0.02179 | 2.6 | 0.00227 | 0.3 | 0.00012 | 0.0 | 0.30319 | 36.7 | 0.00024 | 0.0 | 0.00072 | 0.1 | 0.00475 | 0.6 | - | - |
| 2019 | 0.10499 | 12.4 | 0.09893 | 11.7 | 0.00718 | 0.8 | 0.06154 | 7.3 | 0.03536 | 4.2 | 0.01059 | 1.3 | 0.00111 | 0.1 | 0.01070 | 1.3 | 0.18291 | 21.6 | 0.02284 | 2.7 | 0.00204 | 0.2 | 0.00021 | 0.0 | 0.30174 | 35.6 | 0.00012 | 0.0 | 0.00105 | 0.1 | 0.00521 | 0.6 | - | - |
| 2020 | 0.10500 | 12.4 | 0.10731 | 12.6 | 0.00443 | 0.5 | 0.05596 | 6.6 | 0.03643 | 4.3 | 0.01105 | 1.3 | 0.00082 | 0.1 | 0.00584 | 0.7 | 0.18221 | 21.4 | 0.02053 | 2.4 | 0.00203 | 0.2 | 0.00014 | 0.0 | 0.31411 | 37.0 | 0.00023 | 0.0 | 0.00055 | 0.1 | 0.00326 | 0.4 | - | - |
| 2021 | - | 12.6 | - | 13.8 | - | 0.6 | - | 6.9 | - | 4.2 | - | 1.1 | - | 0.1 | - | 0.6 | - | 22.4 | - | 2.3 | - | 0.3 | - | 0.0 | - | 34.4 | - | 0.0 | - | 0.1 | - | 0.5 | - | - |
