# Supplementary material for: Trends in dental expenditures in Japan with a universal health insurance system
Source: PLoS One. 2023 Oct 5;18(10):e0292547. doi: 10.1371/journal.pone.0292547 (PMC10553203; doi:10.1371/journal.pone.0292547)
Supplement: S5 Table — (DOCX) [file pone.0292547.s005.docx]

**S5 Table. Amount and proportion of services per year for people aged 65 years or older**

| **Year** | **Initial- and repeat-consultation fee (A)** | | **Medical management (B)** | | **At-home treatment (C)** | | **Tests (D)** | | **Diagnostic imaging (E)** | | **Drug administration (F)** | | **Injection (G)** | | **Rehabilitation (H)** | | **Treatment (I)** | | **Surgery (J)** | | **Anaesthesia (K)** | | **Radiotherapy (L)** | | **Crown restoration and prosthesis (M)** | | **Orthodontic treatment (N)** | | **Pathological diagnosis (O)** | | **Hospitalisation fee** | | **Others** | |
| --- | --- | --- | --- | --- | --- | --- | --- | --- | --- | --- | --- | --- | --- | --- | --- | --- | --- | --- | --- | --- | --- | --- | --- | --- | --- | --- | --- | --- | --- | --- | --- | --- | --- | --- |
|  | **Amount** | **%** | **Amount** | **%** | **Amount** | **%** | **Amount** | **%** | **Amount** | **%** | **Amount** | **%** | **Amount** | **%** | **Amount** | **%** | **Amount** | **%** | **Amount** | **%** | **Amount** | **%** | **Amount** | **%** | **Amount** | **%** | **Amount** | **%** | **Amount** | **%** | **Amount** | **%** | **Amount** | **%** |
|  | **(1 trillion yen [≈ 10 billion US dollars])** |  | **(1 trillion yen [≈ 10 billion US dollars])** |  | **(1 trillion yen [≈ 10 billion US dollars])** |  | **(1 trillion yen [≈ 10 billion US dollars])** |  | **(1 trillion yen [≈ 10 billion US dollars])** |  | **(1 trillion yen [≈ 10 billion US dollars])** |  | **(1 trillion yen [≈ 10 billion US dollars])** |  | **(1 trillion yen [≈ 10 billion US dollars])** |  | **(1 trillion yen [≈ 10 billion US dollars])** |  | **(1 trillion yen [≈ 10 billion US dollars])** |  | **(1 trillion yen [≈ 10 billion US dollars])** |  | **(1 trillion yen [≈ 10 billion US dollars])** |  | **(1 trillion yen [≈ 10 billion US dollars])** |  | **(1 trillion yen [≈ 10 billion US dollars])** |  | **(1 trillion yen [≈ 10 billion US dollars])** |  | **(1 trillion yen [≈ 10 billion US dollars])** |  | **(1 trillion yen [≈ 10 billion US dollars])** |  |
| 1996 | 0.04930 | 9.5 | 0.02422 | 4.7 | 0.00604 | 1.2 | 0.01447 | 2.8 | 0.01215 | 2.3 | 0.00830 | 1.6 | 0.00100 | 0.2 | 0.00002 | 0.0 | 0.06060 | 11.6 | 0.01870 | 3.6 | 0.00076 | 0.1 | 0.00040 | 0.1 | 0.32107 | 61.7 | 0.00000 | 0.0 | - | - | 0.00360 | 0.7 | 0.00000 | 0.0 |
| 1997 | 0.05003 | 9.3 | 0.02855 | 5.3 | 0.00660 | 1.2 | 0.01533 | 2.8 | 0.01304 | 2.4 | 0.00843 | 1.6 | 0.00100 | 0.2 | 0.00003 | 0.0 | 0.06413 | 11.9 | 0.01999 | 3.7 | 0.00088 | 0.2 | 0.00017 | 0.0 | 0.32746 | 60.8 | 0.00005 | 0.0 | - | - | 0.00322 | 0.6 | 0.00000 | 0.0 |
| 1998 | 0.05533 | 9.7 | 0.03378 | 5.9 | 0.00962 | 1.7 | 0.01597 | 2.8 | 0.01360 | 2.4 | 0.00823 | 1.4 | 0.00085 | 0.1 | 0.00012 | 0.0 | 0.07330 | 12.8 | 0.02083 | 3.6 | 0.00069 | 0.1 | 0.00033 | 0.1 | 0.33409 | 58.5 | 0.00000 | 0.0 | - | - | 0.00417 | 0.7 | 0.00000 | 0.0 |
| 1999 | 0.06271 | 9.8 | 0.03923 | 6.2 | 0.01092 | 1.7 | 0.01827 | 2.9 | 0.01688 | 2.6 | 0.01045 | 1.6 | 0.00079 | 0.1 | 0.00006 | 0.0 | 0.07746 | 12.1 | 0.02308 | 3.6 | 0.00098 | 0.2 | 0.00014 | 0.0 | 0.37265 | 58.4 | 0.00000 | 0.0 | - | - | 0.00423 | 0.7 | 0.00000 | 0.0 |
| 2000 | 0.06734 | 10.2 | 0.04327 | 6.5 | 0.00767 | 1.2 | 0.01782 | 2.7 | 0.01427 | 2.2 | 0.01026 | 1.5 | 0.00093 | 0.1 | 0.00016 | 0.0 | 0.06790 | 10.2 | 0.02798 | 4.2 | 0.00101 | 0.2 | 0.00007 | 0.0 | 0.39920 | 60.2 | 0.00000 | 0.0 | - | - | 0.00503 | 0.8 | 0.00000 | 0.0 |
| 2001 | 0.06691 | 9.4 | 0.04808 | 6.8 | 0.01031 | 1.5 | 0.01856 | 2.6 | 0.01679 | 2.4 | 0.01177 | 1.7 | 0.00092 | 0.1 | 0.00016 | 0.0 | 0.07960 | 11.2 | 0.02504 | 3.5 | 0.00092 | 0.1 | 0.00016 | 0.0 | 0.42668 | 60.1 | 0.00000 | 0.0 | - | - | 0.00444 | 0.6 | 0.00000 | 0.0 |
| 2002 | 0.07523 | 10.6 | 0.04298 | 6.1 | 0.01332 | 1.9 | 0.02981 | 4.2 | 0.01775 | 2.5 | 0.01094 | 1.5 | 0.00075 | 0.1 | 0.00022 | 0.0 | 0.07896 | 11.1 | 0.02574 | 3.6 | 0.00108 | 0.2 | 0.00015 | 0.0 | 0.40584 | 57.3 | 0.00000 | 0.0 | - | - | 0.00601 | 0.8 | 0.00000 | 0.0 |
| 2003 | 0.08132 | 11.3 | 0.04535 | 6.3 | 0.02586 | 3.6 | 0.02923 | 4.1 | 0.01660 | 2.3 | 0.01188 | 1.7 | 0.00101 | 0.1 | 0.00011 | 0.0 | 0.08163 | 11.4 | 0.02669 | 3.7 | 0.00101 | 0.1 | 0.00011 | 0.0 | 0.39033 | 54.4 | 0.00000 | 0.0 | - | - | 0.00604 | 0.8 | 0.00000 | 0.0 |
| 2004 | 0.08848 | 11.5 | 0.05268 | 6.9 | 0.02102 | 2.7 | 0.03075 | 4.0 | 0.01879 | 2.4 | 0.01324 | 1.7 | 0.00096 | 0.1 | 0.00005 | 0.0 | 0.08667 | 11.3 | 0.02385 | 3.1 | 0.00096 | 0.1 | 0.00030 | 0.0 | 0.42432 | 55.3 | 0.00009 | 0.0 | - | - | 0.00550 | 0.7 | 0.00000 | 0.0 |
| 2005 | 0.09993 | 12.4 | 0.05764 | 7.2 | 0.01248 | 1.6 | 0.03378 | 4.2 | 0.01950 | 2.4 | 0.01341 | 1.7 | 0.00095 | 0.1 | 0.00013 | 0.0 | 0.10035 | 12.5 | 0.02389 | 3.0 | 0.00147 | 0.2 | 0.00019 | 0.0 | 0.43611 | 54.2 | 0.00000 | 0.0 | - | - | 0.00530 | 0.7 | 0.00000 | 0.0 |
| 2006 | 0.08233 | 10.0 | 0.06881 | 8.4 | 0.01618 | 2.0 | 0.04094 | 5.0 | 0.02142 | 2.6 | 0.01242 | 1.5 | 0.00089 | 0.1 | 0.00009 | 0.0 | 0.10407 | 12.7 | 0.02386 | 2.9 | 0.00109 | 0.1 | 0.00021 | 0.0 | 0.44202 | 53.9 | 0.00000 | 0.0 | - | - | 0.00598 | 0.7 | 0.00000 | 0.0 |
| 2007 | 0.08054 | 9.8 | 0.06265 | 7.6 | 0.01429 | 1.7 | 0.04022 | 4.9 | 0.02123 | 2.6 | 0.01403 | 1.7 | 0.00089 | 0.1 | 0.00011 | 0.0 | 0.11341 | 13.8 | 0.02407 | 2.9 | 0.00106 | 0.1 | 0.00019 | 0.0 | 0.44557 | 54.1 | 0.00000 | 0.0 | - | - | 0.00593 | 0.7 | 0.00000 | 0.0 |
| 2008 | 0.08902 | 10.2 | 0.10092 | 11.6 | 0.02120 | 2.4 | 0.04185 | 4.8 | 0.02279 | 2.6 | 0.01408 | 1.6 | 0.00111 | 0.1 | 0.00023 | 0.0 | 0.11466 | 13.1 | 0.02411 | 2.8 | 0.00149 | 0.2 | 0.00043 | 0.0 | 0.43193 | 49.5 | 0.00000 | 0.0 | 0.00069 | 0.1 | 0.00810 | 0.9 | - | - |
| 2009 | 0.08440 | 9.3 | 0.10369 | 11.5 | 0.04378 | 4.8 | 0.04421 | 4.9 | 0.02356 | 2.6 | 0.01449 | 1.6 | 0.00088 | 0.1 | 0.00022 | 0.0 | 0.11289 | 12.5 | 0.02643 | 2.9 | 0.00130 | 0.1 | 0.00030 | 0.0 | 0.43850 | 48.5 | 0.00000 | 0.0 | 0.00043 | 0.0 | 0.00880 | 1.0 | - | - |
| 2010 | 0.09725 | 10.3 | 0.11082 | 11.7 | 0.04683 | 5.0 | 0.04542 | 4.8 | 0.02447 | 2.6 | 0.01515 | 1.6 | 0.00081 | 0.1 | 0.00051 | 0.1 | 0.12614 | 13.3 | 0.02423 | 2.6 | 0.00134 | 0.1 | 0.00034 | 0.0 | 0.44283 | 46.8 | 0.00000 | 0.0 | 0.00072 | 0.1 | 0.00871 | 0.9 | - | - |
| 2011 | 0.10432 | 10.5 | 0.12531 | 12.7 | 0.05485 | 5.5 | 0.05034 | 5.1 | 0.02470 | 2.5 | 0.01399 | 1.4 | 0.00084 | 0.1 | 0.00047 | 0.0 | 0.13240 | 13.4 | 0.02633 | 2.7 | 0.00141 | 0.1 | 0.00045 | 0.0 | 0.44602 | 45.0 | 0.00000 | 0.0 | 0.00049 | 0.0 | 0.00825 | 0.8 | - | - |
| 2012 | 0.10790 | 10.4 | 0.11026 | 10.6 | 0.06497 | 6.3 | 0.05064 | 4.9 | 0.02677 | 2.6 | 0.01472 | 1.4 | 0.00025 | 0.0 | 0.00063 | 0.1 | 0.15147 | 14.6 | 0.02777 | 2.7 | 0.00140 | 0.1 | 0.00028 | 0.0 | 0.47190 | 45.5 | 0.00001 | 0.0 | 0.00050 | 0.0 | 0.00789 | 0.8 | - | - |
| 2013 | 0.11558 | 10.6 | 0.12183 | 11.2 | 0.05479 | 5.0 | 0.05510 | 5.1 | 0.02799 | 2.6 | 0.01399 | 1.3 | 0.00027 | 0.0 | 0.00081 | 0.1 | 0.17014 | 15.7 | 0.02932 | 2.7 | 0.00165 | 0.2 | 0.00024 | 0.0 | 0.48595 | 44.7 | 0.00000 | 0.0 | 0.00081 | 0.1 | 0.00869 | 0.8 | - | - |
| 2014 | 0.12049 | 11.0 | 0.09742 | 8.9 | 0.06183 | 5.6 | 0.05710 | 5.2 | 0.02941 | 2.7 | 0.01407 | 1.3 | 0.00019 | 0.0 | 0.03121 | 2.8 | 0.16544 | 15.1 | 0.02805 | 2.6 | 0.00128 | 0.1 | 0.00041 | 0.0 | 0.48316 | 44.0 | 0.00000 | 0.0 | 0.00072 | 0.1 | 0.00809 | 0.7 | - | - |
| 2015 | 0.12201 | 10.8 | 0.10670 | 9.5 | 0.07101 | 6.3 | 0.06064 | 5.4 | 0.03127 | 2.8 | 0.01490 | 1.3 | 0.00137 | 0.1 | 0.03322 | 2.9 | 0.17200 | 15.3 | 0.02806 | 2.5 | 0.00157 | 0.1 | 0.00033 | 0.0 | 0.47413 | 42.1 | 0.00001 | 0.0 | 0.00083 | 0.1 | 0.00865 | 0.8 | - | - |
| 2016 | 0.12386 | 10.8 | 0.10697 | 9.3 | 0.07382 | 6.4 | 0.06212 | 5.4 | 0.03243 | 2.8 | 0.01441 | 1.3 | 0.00148 | 0.1 | 0.03335 | 2.9 | 0.18101 | 15.8 | 0.02846 | 2.5 | 0.00169 | 0.1 | 0.00035 | 0.0 | 0.47821 | 41.6 | 0.00001 | 0.0 | 0.00093 | 0.1 | 0.00922 | 0.8 | - | - |
| 2017 | 0.12599 | 10.8 | 0.11083 | 9.5 | 0.07757 | 6.6 | 0.06302 | 5.4 | 0.03342 | 2.9 | 0.01442 | 1.2 | 0.00166 | 0.1 | 0.03371 | 2.9 | 0.19168 | 16.4 | 0.02824 | 2.4 | 0.00184 | 0.2 | 0.00031 | 0.0 | 0.47364 | 40.6 | 0.00001 | 0.0 | 0.00097 | 0.1 | 0.00961 | 0.8 | - | - |
| 2018 | 0.12597 | 10.5 | 0.11643 | 9.7 | 0.08245 | 6.9 | 0.06373 | 5.3 | 0.03464 | 2.9 | 0.01384 | 1.2 | 0.00197 | 0.2 | 0.03525 | 3.0 | 0.20158 | 16.9 | 0.02930 | 2.5 | 0.00190 | 0.2 | 0.00038 | 0.0 | 0.47572 | 39.8 | 0.00001 | 0.0 | 0.00104 | 0.1 | 0.01044 | 0.9 | - | - |
| 2019 | 0.14838 | 12.2 | 0.12121 | 10.0 | 0.08931 | 7.3 | 0.05410 | 4.4 | 0.03400 | 2.8 | 0.01247 | 1.0 | 0.00147 | 0.1 | 0.03409 | 2.8 | 0.18460 | 15.2 | 0.02680 | 2.2 | 0.00216 | 0.2 | 0.00040 | 0.0 | 0.49818 | 40.9 | 0.00010 | 0.0 | 0.00114 | 0.1 | 0.00948 | 0.8 | - | - |
| 2020 | 0.12853 | 10.9 | 0.14200 | 12.0 | 0.08346 | 7.1 | 0.05689 | 4.8 | 0.03361 | 2.8 | 0.01471 | 1.2 | 0.00242 | 0.2 | 0.03519 | 3.0 | 0.19674 | 16.6 | 0.02786 | 2.4 | 0.00212 | 0.2 | 0.00059 | 0.0 | 0.44865 | 37.9 | 0.00003 | 0.0 | 0.00092 | 0.1 | 0.00927 | 0.8 | - | - |
| 2021 | - | 10.8 | - | 12.8 | - | 7.3 | - | 4.9 | - | 2.7 | - | 1.1 | - | 0.2 | - | 2.9 | - | 17.3 | - | 2.2 | - | 0.2 | - | 0.0 | - | 36.9 | - | 0.0 | - | 0.1 | - | 0.7 | - | - |
