# Supplementary material for: Trends in dental expenditures in Japan with a universal health insurance system
Source: PLoS One. 2023 Oct 5;18(10):e0292547. doi: 10.1371/journal.pone.0292547 (PMC10553203; doi:10.1371/journal.pone.0292547)
Supplement: S6 Table — (DOCX) [file pone.0292547.s006.docx]

**S6 Table. Amount and proportion of dental materials for restoration per year**

| **Year** | **All materials for restoration** | | | **Dental gold-silver-palladium alloys** | | | **Other dental metal materials** | | | **Dental non-metal materials** | | | **Other materials** | | |
| --- | --- | --- | --- | --- | --- | --- | --- | --- | --- | --- | --- | --- | --- | --- | --- |
|  | **Amount** | **% in total** | **% in the M category** | **Amount** | **% in total** | **% in the M category** | **Amount** | **% in total** | **% in the M category** | **Amount** | **% in total** | **% in the M category** | **Amount** | **% in total** | **% in the M category** |
|  | **(1 trillion yen [≈ 10 billion US dollars])** |  |  | **(1 trillion yen [≈ 10 billion US dollars])** |  |  | **(1 trillion yen [≈ 10 billion US dollars])** |  |  | **(1 trillion yen [≈ 10 billion US dollars])** |  |  | **(1 trillion yen [≈ 10 billion US dollars])** |  |  |
| 1996 | 0.16337 | 6.2 | 12.3 | 0.09423 | 3.6 | 7.1 | 0.01240 | 0.5 | 0.9 | 0.02850 | 1.1 | 2.1 | 0.02824 | 1.1 | 2.1 |
| 1997 | 0.18087 | 7.0 | 13.9 | 0.11436 | 4.4 | 8.8 | 0.01208 | 0.5 | 0.9 | 0.02678 | 1.0 | 2.1 | 0.02766 | 1.1 | 2.1 |
| 1998 | 0.14806 | 5.8 | 11.8 | 0.08345 | 3.3 | 6.7 | 0.01203 | 0.5 | 1.0 | 0.02436 | 1.0 | 1.9 | 0.02821 | 1.1 | 2.3 |
| 1999 | 0.14879 | 5.7 | 12.2 | 0.08150 | 3.1 | 6.7 | 0.01198 | 0.5 | 1.0 | 0.02772 | 1.1 | 2.3 | 0.02760 | 1.1 | 2.3 |
| 2000 | 0.14698 | 5.6 | 11.2 | 0.08603 | 3.3 | 6.5 | 0.01252 | 0.5 | 1.0 | 0.02398 | 0.9 | 1.8 | 0.02445 | 0.9 | 1.9 |
| 2001 | 0.20152 | 7.5 | 14.5 | 0.13719 | 5.1 | 9.9 | 0.01312 | 0.5 | 0.9 | 0.02582 | 1.0 | 1.9 | 0.02540 | 0.9 | 1.8 |
| 2002 | 0.16061 | 5.9 | 12.3 | 0.10477 | 3.9 | 8.0 | 0.00975 | 0.4 | 0.7 | 0.02378 | 0.9 | 1.8 | 0.02232 | 0.8 | 1.7 |
| 2003 | 0.15035 | 5.7 | 11.8 | 0.09721 | 3.7 | 7.7 | 0.00783 | 0.3 | 0.6 | 0.02430 | 0.9 | 1.9 | 0.02101 | 0.8 | 1.7 |
| 2004 | 0.11843 | 4.5 | 9.7 | 0.06513 | 2.5 | 5.3 | 0.00833 | 0.3 | 0.7 | 0.02305 | 0.9 | 1.9 | 0.02192 | 0.8 | 1.8 |
| 2005 | 0.12359 | 4.6 | 10.1 | 0.06894 | 2.5 | 5.6 | 0.00821 | 0.3 | 0.7 | 0.02580 | 1.0 | 2.1 | 0.02064 | 0.8 | 1.7 |
| 2006 | 0.12148 | 4.6 | 10.2 | 0.06882 | 2.6 | 5.8 | 0.00920 | 0.4 | 0.8 | 0.02556 | 1.0 | 2.1 | 0.01790 | 0.7 | 1.5 |
| 2007 | 0.14400 | 5.5 | 11.9 | 0.09032 | 3.5 | 7.5 | 0.00907 | 0.3 | 0.7 | 0.02770 | 1.1 | 2.3 | 0.01691 | 0.6 | 1.4 |
| 2008 | 0.14674 | 5.5 | 12.9 | 0.09398 | 3.5 | 8.2 | 0.00806 | 0.3 | 0.7 | 0.02828 | 1.1 | 2.5 | 0.01641 | 0.6 | 1.4 |
| 2009 | 0.14313 | 5.3 | 12.7 | 0.09092 | 3.4 | 8.1 | 0.00765 | 0.3 | 0.7 | 0.02896 | 1.1 | 2.6 | 0.01561 | 0.6 | 1.4 |
| 2010 | 0.13515 | 4.9 | 12.1 | 0.08414 | 3.1 | 7.5 | 0.00834 | 0.3 | 0.7 | 0.02671 | 1.0 | 2.4 | 0.01595 | 0.6 | 1.4 |
| 2011 | 0.17131 | 6.1 | 15.2 | 0.11831 | 4.2 | 10.5 | 0.00847 | 0.3 | 0.8 | 0.02862 | 1.0 | 2.5 | 0.01591 | 0.6 | 1.4 |
| 2012 | 0.18971 | 6.6 | 16.3 | 0.13788 | 4.8 | 11.9 | 0.00789 | 0.3 | 0.7 | 0.02916 | 1.0 | 2.5 | 0.01478 | 0.5 | 1.3 |
| 2013 | 0.18301 | 6.3 | 16.1 | 0.13271 | 4.6 | 11.7 | 0.00768 | 0.3 | 0.7 | 0.02780 | 1.0 | 2.5 | 0.01482 | 0.5 | 1.3 |
| 2014 | 0.18211 | 6.4 | 16.5 | 0.12879 | 4.5 | 11.7 | 0.00871 | 0.3 | 0.8 | 0.02983 | 1.0 | 2.7 | 0.01476 | 0.5 | 1.3 |
| 2015 | 0.19689 | 6.8 | 18.1 | 0.14252 | 4.9 | 13.1 | 0.00808 | 0.3 | 0.7 | 0.03263 | 1.1 | 3.0 | 0.01366 | 0.5 | 1.3 |
| 2016 | 0.20239 | 6.9 | 18.6 | 0.14994 | 5.1 | 13.8 | 0.00548 | 0.2 | 0.5 | 0.03395 | 1.2 | 3.1 | 0.01301 | 0.4 | 1.2 |
| 2017 | 0.18581 | 6.3 | 17.5 | 0.13380 | 4.5 | 12.6 | 0.00513 | 0.2 | 0.5 | 0.03408 | 1.2 | 3.2 | 0.01280 | 0.4 | 1.2 |
| 2018 | 0.19656 | 6.6 | 18.6 | 0.14346 | 4.8 | 13.6 | 0.00485 | 0.2 | 0.5 | 0.03586 | 1.2 | 3.4 | 0.01239 | 0.4 | 1.2 |
| 2019 | 0.18208 | 6.0 | 18.2 | 0.12637 | 4.2 | 12.6 | 0.00443 | 0.1 | 0.4 | 0.03973 | 1.3 | 4.0 | 0.01155 | 0.4 | 1.2 |
| 2020 | 0.22793 | 7.6 | 21.8 | 0.17312 | 5.8 | 16.6 | 0.00497 | 0.2 | 0.5 | 0.03864 | 1.3 | 3.7 | 0.01120 | 0.4 | 1.1 |
| 2021 | - | 7.9 | 24.3 | - | 6.1 | 18.9 | - | 0.2 | 0.5 | - | 1.3 | 3.9 | - | 0.3 | 1.0 |
