# Supplementary material for: Trends in dental expenditures in Japan with a universal health insurance system
Source: PLoS One. 2023 Oct 5;18(10):e0292547. doi: 10.1371/journal.pone.0292547 (PMC10553203; doi:10.1371/journal.pone.0292547)
Supplement: Attachment — Submitted filename: Response to Reviewers.docx [file pone.0292547.s007.docx]

**RESPONSES TO REVIEWERS’ COMMENTS**

Dear Ahmed Jamleh, Academic Editor, and the reviewer

Thank you for giving me the opportunity to submit a revised draft of our manuscript. We appreciate the time and effort that the editor and reviewer have invested in offering valuable feedback. We have addressed each comment by providing point-by-point responses and have made corresponding changes in the manuscript.

**Journal requirements**

Thank you for checking the format of our manuscript. Following your comments, we have corrected all files to conform to the PLOS ONE format. Please see below.

**1.** Please ensure that your manuscript meets PLOS ONE's style requirements, including those for file naming. The PLOS ONE style templates can be found at

https://journals.plos.org/plosone/s/file?id=wjVg/PLOSOne_formatting_sample_main_body.pdf

and

https://journals.plos.org/plosone/s/file?id=ba62/PLOSOne_formatting_sample_title_authors_affiliations.pdf

**RESPONSE**

We have modified our manuscript files to adhere to the style requirements of PLOS ONE.

**2.** Please ensure that you have specified (1) whether consent was informed and (2) what type you obtained (for instance, written or verbal, and if verbal, how it was documented and witnessed). If your study included minors, state whether you obtained consent from parents or guardians. If the need for consent was waived by the ethics committee, please include this information.

**RESPONSE**

The datasets used in this study are obtained from official national surveys conducted by the government, and these datasets are publicly available. Additionally, the datasets do not contain any personal information, so we believe that the ethical concerns you mentioned are not applicable to our study. We have added the explanation in our manuscript that we used publicly available secondary datasets provided by the government.

**(Line 135–138, page 7)**

**Ethical Approval**

This study used publicly available datasets obtained from official national surveys conducted by the government of Japan. The datasets do not contain any personal information; therefore, ethical approval was not required.

**3.** Thank you for stating the following financial disclosure:

“This study was supported by a grant from the FUTOKU Foundation in 2022 for research projects.”

Please state what role the funders took in the study. If the funders had no role, please state: "The funders had no role in study design, data collection and analysis, decision to publish, or preparation of the manuscript."

If this statement is not correct you must amend it as needed.

Please include this amended Role of Funder statement in your cover letter; we will change the online submission form on your behalf.

**RESPONSE**

The funder has no role; therefore, we have stated that "The funders had no role in study design, data collection and analysis, decision to publish, or preparation of the manuscript." in the cover letter.

**4.** Please include captions for your Supporting Information files at the end of your manuscript, and update any in-text citations to match accordingly. Please see our Supporting Information guidelines for more information: http://journals.plos.org/plosone/s/supporting-information.

**RESPONSE**

Following the guidelines, we have added and updated the captions and citations of the supporting information file at the end of the manuscript.

**Editor**

Thank you for your valuable feedback and suggestions. Please find below our point-by-point response to your comments and concerns.

We added the information about expenditures in 2020 from the Estimates of National Medical Care Expenditure, which were made available by the government on November 30th, 2022. As a result, we have updated the tables and figures, but these changes were only made to the results from 2020. We have not made any changes to the other results.

**1.** Rearrange the sequence of references. In P3, I see reference no.25 comes after citing the first 9 references.

**RESPONSE**

Sorry for our mistake. We have re-confirmed all the references and updated them.

**2.** P6: You stated that dental procedures are classified in 14 areas but based on what you wrote (A-O), they are supposed to be 15! Please check.

**RESPONSE**

We appreciate your comment regarding the category number, which is now correctly indicated as 15.

**(Line 106–107, page 6)**

The MHLW classified dental procedures into 15 areas:

**3.** In the reference list, kindly update the access date after checking their availability.

**RESPONSE**

We have updated and confirmed the references and their availability.

**Reviewer 1**

We appreciate the time and effort that you have invested in reviewing our manuscript. We have responded to all comments carefully, as shown below.

We added the information about expenditures in 2020 from the Estimates of National Medical Care Expenditure, which were made available by the government on November 30th, 2022. As a result, we have updated the tables and figures, but these changes were only made to the results from 2020. We have not made any changes to the other results.

**1.** Abstract: medical management category should be explicit.

**RESPONSE**

We have added the explanation of "medical management" category in the abstract.

**(Line 18–20, page 2)**

In 0-14 years persons, expenses on the crown restoration and prosthesis category decreased while the medical management category (mainly including fees for a management plan for oral diseases or oral functions) increased.

**2.** Abstract: The conclusion section should have the main results in quantitative statements as well.

**RESPONSE**

We have revised the conclusion section based on the quantitative results.

**(Line 23–26, page 2–3)**

**Conclusion**

The amount of dental spending in Japan substantially increased from 1.96 trillion yen in 1984 to 3.00 trillion yen in 2020), a 1.53-fold increase. The observed changes in annual dental spending varied across age groups and types of service.

**3.** Page 4, Line 58: You have stated the proportion of older persons who had 20 teeth or more in 1993 and 2016. It is too old. Authors need to report the latest data.

**RESPONSE**

We agree that this information is old. However, due to the pandemic of the COVID-19, the government cancelled the Survey of Dental Diseases that was scheduled for 2021, and as a result, the most recent available national data is from 2016 because the government conducts the survey every five years. Furthermore, there are no national surveys that report the oral health status of the entire Japanese population. Only the DMFT index of 12 years old is available because another national survey collects the health status of schoolchildren.

**4.** This study design did not control or adjust for underlying secular trends, so it may have failed to adequately capture dental expenditure trends. Moreover, it was not able to minimize threats to internal validity, including random fluctuations, history, and maturation; these extraneous events and changes over time.

**RESPONSE**

We agree with you. Although the Estimates of National Medical Care Expenditure covers all the expenditures of insurance services in Japan, as this descriptive study did not consider potentially underlying secular trends, the trends we found in this study might be biased. Therefore, we have toned down the expressions of the discussion and stated the limitations in the discussion section.

**(Line 204–209, page 11)**

First, the datasets of SECHC were June data and not annual data [23]. Therefore, the proportions of each type of dental service have random errors. Furthermore, as the sampling method has changed since 2015 [23], the data before 2014 have potentially increased the random errors. Moreover, this study did not account for underlying secular trends and extraneous factors. Therefore, these limitations can violate internal validity.

**5.** Your references and literature search shows that studies have been conducted on the trend in dental expenditures under the universal health insurance system in Japan. Please highlight what additional value your study adds and the justification for your study.

**RESPONSE**

We have emphasized the novelty of this study compared to previous studies.

**(Line 37–42, page 3)**

Previous studies on dental expenditures in Japan have primarily focused on total costs without examining the spending pattern across different age groups, types of services, and over time. A detailed examination of trends in dental expenditures is crucial for the development of future sustainable plans for the allocation of financial resources. By understanding the specific patterns and trends in dental expenditures, policymakers can make decisions to ensure effective distribution of resources in the dental healthcare sector.
