# Supplementary material for: Trends in dental expenditures in Japan with a universal health insurance system
Source: PLoS One. 2023 Oct 5;18(10):e0292547. doi: 10.1371/journal.pone.0292547 (PMC10553203; doi:10.1371/journal.pone.0292547)
Supplement: Attachment — Submitted filename: Response to Reviewers.docx [file pone.0292547.s008.docx]

**RESPONSES TO REVIEWERS’ COMMENTS**

Dear Professor Ahmed Jamleh,

We would like to thank you for your insightful comment on our paper. We have responded to the comment and made changes to the manuscript accordingly.

**Journal requirements**

**1.** Please review your reference list to ensure that it is complete and correct. If you have cited papers that have been retracted, please include the rationale for doing so in the manuscript text, or remove these references and replace them with relevant current references. Any changes to the reference list should be mentioned in the rebuttal letter that accompanies your revised manuscript. If you need to cite a retracted article, indicate the article’s retracted status in the References list and also include a citation and full reference for the retraction notice.

**RESPONSE**

Thank you for checking the references in our manuscript. We have confirmed that there was no retracted reference. We have made minor revisions to the citation information. In addition, following the editor's comment, a new reference has been added.

*"2. Sakamoto H, Rahman M, Nomura S, Okamoto E, Koike S, Yasunaga H, et al. Japan health system review. New Delhi: World Health Organization. Regional Office for South-East Asia; 2018. Available: https://apps.who.int/iris/handle/10665/259941"*

was revised to

*"2. Sakamoto H, Rahman M, Nomura S, Okamoto E, Koike S, Yasunaga H, et al. Japan health system review. Health Syst Transit. 2018;8. Available: https://apps.who.int/iris/handle/10665/259941"*

*"5. OECD. Health at a Glance 2021. 2021. doi:https://doi.org/https://doi.org/10.1787/ae3016b9-en"*

was revised to

*"5. OECD. Health at a Glance 2021. 2021. Available: https://www.oecd-ilibrary.org/content/publication/ae3016b9-en"*

*"7. OECD. Demography: Population. In: OECD Data [Internet]. [cited 17 Nov 2022]. Available: http://data.oecd.org/pop/population.htm"*

was changed to

*"7. Statistics Bureau of Japan. Population estimates in Japan (in Japanese). 2022 [cited 25 Feb 2022]. Available: https://www.stat.go.jp/data/jinsui/2.html"*

*"9. Brennan D. Dental Health Services Epidemiology. In: Peres MA, Antunes JLF, Watt RG, editors. Oral Epidemiology: A Textbook on Oral Health Conditions, Research Topics and Methods. Cham: Springer International Publishing; 2021. pp. 395–407. doi:10.1007/978-3-030-50123-5_26"*

was added.

*"9. Japan Health Policy NOW. Japanese Health Policy. [cited 17 Nov 2022]. Available: http://japanhpn.org/en/_japan_healthpolicy/"*

was revised to

*"11. Health and Global Policy Institute. Japanese Health Policy. 2019 [cited 17 Nov 2022]. Available: http://japanhpn.org/en/_japan_healthpolicy/"*

*"13. Kamijo H. Reform of the Social Security System and Prospects for Oral Health and Care in Japan -Influence of daily dental treatment and management of dental clinics- (in Japanese). SHIKWA GAKUHO. 2017;117: 1–16. doi:10.15041/tdcgakuho.117.1"*

was revised to

*"15. Kamijo H. Reform of the Social Security System and Prospects for Oral Health and Care in Japan -Influence of daily dental treatment and management of dental clinics- (in Japanese). J Tokyo Dent Coll Soc. 2017;117: 1–16. doi:10.15041/tdcgakuho.117.1"*

*"20. Nakai M, Niinomi M. Chapter 12 Dental Metallic Materials. Advances in Metallic Biomaterials: Processing and Applications. Springer; 2015."*

was revised to

*"20. Nakai M, Niinomi M. Dental Metallic Materials. In: Niinomi M, Narushima T, Nakai M, editors. Advances in Metallic Biomaterials. Berlin, Heidelberg: Springer; 2015. pp. 251–281. doi:10.1007/978-3-662-46842-5_12*

*"24. Statistics Bureau, Ministry of Internal Affairs and Communications. Consumer Price Index (in Japanese). In: Consumer Price Index [Internet]. [cited 14 Jun 2022]. Available: https://www.stat.go.jp/data/cpi/"*

was revised to

*"24. Statistics Bureau, Ministry of Internal Affairs and Communications. Consumer Price Index (in Japanese). [cited 20 Sep 2023]. Available: https://www.stat.go.jp/data/cpi/"*

**Editor**

**1.** L37-39: Please consider adding references. With this, you need to consider rearranging the references

**RESPONSE**

We wish to thank the editor for this comment. We have added references; thus, all the references have been rearranged.

**(Line 37–43, page 3)**

Previous studies on dental expenditures in Japan have primarily focused on total costs without examining the spending pattern across different age groups, types of services, and over time [3,8]. A detailed examination of trends in dental expenditures is crucial for the development of future sustainable plans for the allocation of financial resources [9]. By understanding the specific patterns and trends in dental expenditures, policymakers can make decisions to ensure effective distribution of resources in the dental healthcare sector [9,10].
